# The right immune-modulation at the right time: thymosin α1 for prevention of severe COVID-19 in cancer patients

**DOI:** 10.2217/fon-2020-0754

**Published:** 2021-02-04

**Authors:** Melissa Bersanelli, Diana Giannarelli, Alessandro Leonetti, Sebastiano Buti, Marcello Tiseo, Antonio Nouvenne, Andrea Ticinesi, Tiziana Meschi, Giuseppe Procopio, Riccardo Danielli

**Affiliations:** ^1^Medical Oncology Unit, University Hospital of Parma, Via Gramsci 14, Parma, 43126, Italy; ^2^Medicine & Surgery Department, University of Parma, Via Gramsci 14, Parma, 43126, Italy; ^3^Biostatistical Unit, Regina Elena National Cancer Institute, IRCCS, Via Elio Chianesi 53, Rome, 00144, Italy; ^4^Geriatric Rehabilitation Medical Department, University Hospital of Parma, Via Gramsci 14, Parma, 43126, Italy; ^5^Genito-Urinary Oncology Unit, Fondazione IRCCS Istituto Nazionale Tumori of Milan, Via Giacomo Venezian, 1, Milano, 20133, Italy; ^6^Immuno-Oncology Unit, University Hospital of Siena, Viale Mario Bracci 16, Siena, 53100, Italy

**Keywords:** caner, cancer patients, COVID-19, prevention, prophylaxis, SARS-CoV-2, vaccination

## Abstract

We presented the rationale for the use of thymosin α1 as prophylaxis of severe COVID-19 in cancer patients undergoing active treatment, constituting the background for the PROTHYMOS study, a prospective, multicenter, open-label, Phase II randomized study, currently in its start-up phase (Eudract no. 2020-006020-13). We aim to offer new hope for this incurable disease, especially to frail patient population, such as patients with cancer. The hypothesis of an effective prophylactic approach to COVID-19 would have immediate clinical relevance, especially given the lack of curative approaches. Moreover, in the ‘COVID-19 vaccine race era’ both clinical and biological results coming from the PROTHYMOS trials could even support the rationale for future combinatorial approaches, trying to rise vaccine efficacy in frail individuals.

## Lay abstract

We present scientific evidence in favor of using a drug (thymosin-α1) that modulates the immune system functions to try and prevent severe COVID-19 in cancer patients who are currently receiving anticancer treatment. Thymosin-α1 is produced normally by the body in the thymus, which is present in children but not in adults. Given the better outcomes of SARS-CoV-2 infections in children, we thought that thymosin-α1 could help to protect adults from severe infections as well. In this review, we explain some scientific evidence and the background of our clinical trial, PROTHYMOS, which is investigating this preventive treatment. Our aim is to offer a new hope to these at-risk cancer patients, particularly for the elderly who are at most risk of developing severe COVID-19. Given the lack of approaches that can provide cures to COVID-19, any possibility to prevent severe infection should be explored.

## COVID-19 stages: from SARS-CoV-2 infection to the acute respiratory distress syndrome

The evidence about the new disease COVID-19, responsible of the still currently ongoing pandemic, is finally rapidly increasing, ranging from demonstrations of the etiopathogenetic mechanisms, to (up today scarce) clinical data for treating patients with respiratory impairment. In this scenario, some authors have pointed out that the timeline of the disease history is characterized by different stages, the awareness of which is probably needed for a proper therapeutic approach [1]. The use of a three-stage classification system has been proposed, with three grades of increasing clinical severity. The first stage is represented by the early infection, with the viremic phase, during which SARS-CoV-2 binds to its target ACE-2 receptor on human cells and multiplies in the host, initially manifesting with mild upper-tract respiratory symptoms, due to the abundance of ACE-2 receptors in the lung. In the second stage, the pulmonary damage is established, with interstitial pneumonia revealed by computed tomography scans, often showing bilateral ground glass opacities, and leading to progressive respiratory impairment. The most severe phase of COVID-19, occurring in a minority of patients but unfortunately frequently observed worldwide in the last two months, is represented by the systemic hyperinflammation phase, characterized by a typical cytokine release syndrome. In this late stage, inflammation markers are elevated in the serum of patients, becoming responsible of organ damage and failure and of fibrosis of lung tissue. Beyond the typical acute respiratory distress syndrome (ARDS), a multiorgan involvement is frequent, with thromboembolic events, myocarditis, renal and hepatic impairment, risk of cardiopulmonary collapse [1–4].

The therapeutic approach for COVID-19 patients should not disregard this likely reliable timing. Antiviral drugs, potentially effective in the viremic phase (early stage), have recently demonstrated not to provide benefit when used in the later phases of the disease [5]. On the other hand, immunosuppressants like tocilizumab (anti-IL-6 antibody) or the most common corticosteroids (methylprednisolone) have shown a certain benefit, according to recent clinical reports, allowing dominating the cytokine storm that characterizes the late stage of COVID-19 [6–8].

It is likely that the differential effectiveness of such different therapeutic approaches can be due to an immunological shift occurring between the early and the later stages. The initial phase is probably characterized by impaired immunity, with lymphopenia facilitating the increase of the viral load. Indeed, it was recently reported that T cells are decreased and exhausted in patients with COVID-19 [9]. The onset of the respiratory impairment typically occurs during the immunological shift from defective to aberrant response, when an excessive and dysfunctional host immune response leads to ARDS [1].

In the first stage, anti-viral therapies could be beneficial, and immunosuppression could theoretically be dangerous, as it could delay the development of an adequate adaptive immune response. In this light, postexposition prophylaxis protocols should be investigated with caution and carefully considering the different profiles of the drug.

In the second, pulmonary stage, when increasing levels of inflammation (moderate elevation of biomarkers) lead to clinical deterioration and to respiratory impairment, some immunosuppression could be beneficial.

Then, in the cytokine storm phase, more aggressive immunomodulatory treatment is probably needed, to control the hyperinflammatory stage.

## Thymosin α1

Thymosin α1, is a synthetic version of a naturally occurring 28-amino acid peptide acetylated at its amino terminus. Chemically synthesized Tα1 is identical in amino acid sequence to thymosin α1 isolated from thymosin fraction-5, an extract from the thymus gland, which can be administered with subcutaneous injection. Despite its exact mechanism of action is not known, its effect as pleiotropic biological response modifier and as immune cell modulator has been demonstrated [10–15]. Thymosin α1 is expected to have clinical benefits in disorders where immune responses are altered. Of note, it can modulate and balance the immune system in different directions, depending on the immunological status of the host. Its effects have been investigated in clinical studies on various inflammatory and viral diseases, including sepsis, bone marrow transplant-related infections and severe acute respiratory syndromes [12–14].

Thymosin α1 has received marketing approval in close to 40 countries; approved indications include hepatitis B and C, severe sepsis with lymphopenia, vaccine augmentation, adjuvant to chemotherapy or immunotherapy of cancers. The safety and activity of thymosin α1 has been evaluated in both the preclinical and clinical settings. Adverse events have been infrequent and mild, consisting in local discomfort at the injection site and rare instances of erythema, transient muscle atrophy, polyarthralgia with hand edema and rash (<1% drug related adverse events for all indications). No severe adverse events have been reported [16]. Thymosin α1 has also shown promising results in combination with anticancer chemo and immune therapies, and its possible synergistic effect with immune checkpoint inhibitors (ICI) was reported [17,18]. The favorable combination of thymosin α1 with an anti-PD-1 antibody has been already postulated in an experimental setting in which low doses of thymosin α1, while being ineffective alone, increased the efficacy of an anti-PD-1 antibody in the lung metastasis melanoma model [19]. Moreover, several evidence converge in the suggestion that thymosin α1 represents a plausible candidate to improve the safety and the efficacy profile of ICI [20], and, in particular, a preclinical study has shown the protective role of thymosin α1 from intestinal toxicity in a murine model of ICI-induced colitis [21].

The rationale for the use of thymosin α1 in cancer patients was based on the capabilities to enhance immune response, prevent opportunistic infection and counteract the immunosuppressive side effects associated with conventional therapies. Moreover, immune evaluations revealed beneficial effects of thymosin α1 on NK cell activity and CD4^+^ cell number after the suppression induced by chemotherapy [17]. Eventually, it has also been used in infections, improving outcomes of pulmonary cytomegalovirus in renal transplanted patients and in recipients of haploidentical stem cell transplants for hematologic malignancies [12,22]. Its use was associated with increased T-cell counts and earlier appearance of functional pathogen-specific T-cell responses.

Thymosin α1 monotherapy in chronic hepatitis B was effective in suppressing viral replication compared with untreated control or to interferon, as suggested by a meta-analysis of randomized studies (353 patients) showing that, compared with no antiviral treatment, thymosin α1 suppressed viral replication in both HBeAg-positive and HBeAg-negative patients. The odds ratio of the virological response of thymosin over placebo at the end of treatment, 6 months post-treatment and 12 months post-treatment were 0.56 (95% CI: 0.2–1.52), 1.67 (95% CI: 0.83–3.37) and 2.67 (95% CI: 1.25–5.68), respectively. There was an increasing trend of the virological response with time since the cessation of thymosin treatment (p = 0.02) [23].

Moreover, thymosin α1 has been widely investigated even in severe sepsis [24]. A systematic review of 19 randomized controlled trials showed a reduced mortality in sepsis patients receiving thymosin α1 as compared with control group (relative risk, RR: 0.59; 95% CI: 0.45–0.77; p = 0.0001) [25]. Neither serious Tα1 related adverse events nor discontinuation due to tolerability issues have been recorded in such studies.

Along this line, large, randomized trials have demonstrated that thymosin α1 enhances the immunogenicity of influenza vaccines in immunocompromised patients, reducing the number of cases of influenza and symptoms compared with the vaccine alone, without any safety alerts [26]. Given these previous data, this adjuvant compound might be useful in reducing the required vaccine antigen that may be relevant, if massive vaccination programs are to be undertaken in the case of a pandemic threat.

## Rationale & timing for the potential use of thymosin α1 in COVID-19

After further nationwide lock-downs to prevent the second spread of the virus, several countries in Europe are moving to the so called ‘COVID-19 Phase III’, a likely indefinite period during which citizens must learn how to live together with the virus. Along this line, in the next future, the human contacts will progressively increase, and the risk of new outbreaks will never be averted. In this scenario, it is crucial advancing COVID-19 strategies to prevent a new spread of the virus. Currently, more than 90 vaccines are being developed against COVID-19 by research teams in companies and universities worldwide, with the common goal to elicit an immune response against SARS-CoV-2 neutralizing the viral infection. However, the exact immunologic background of COVID-19 is currently under investigation. It has been identified that CD4^+^ and CD8^+^ lymphocytes are significantly lower in severe/critical COVID-19 patients [9]. The phenomenon of lymphocytes depletion (PLD) has demonstrated prognostic implications, including sepsis, pneumonia, ARDS and more recently also COVID-19 [9]. Cytokines, such as IL-10, IL-6 and TNF-α have been suggested to be involved in T-cell reduction during SARS-CoV-2 infection. Furthermore, recent researches demonstrated that surviving T cell during PLD in COVID-19 are functionally exhausted, suggesting that lymphocyte subsets should be analyzed in these patients, in order to early intervene in the negative consequence of PLD and T-cell exhaustion [9,27,28]. Along this line, PLD could be one of the main targets of thymosin α1 in the context of COVID-19, improving the prognosis of severe cases or even before hindering the progression from the viral phase to the severe inflammation stage.

Recent data suggest that early adaptive immune responses might correlate with better clinical outcome in COVID-19 patients [29]. Thymosin α1 could trigger the adaptive immune response, directly enhancing the recognition of infected cells and modulating T-cells activity. Moreover, it can stimulate both innate and adaptive immunity to clear virus and other nonself agents [30–37]. Along with the basic research data, several clinical studies in very different areas, such as sepsis and cancer have indicated thymosin α1 could improve the imbalance of IFN-γ and IL-4 ratio in CD4^+^ T cell and adjust the immune state [24,38].

The pleiotropic function of thymosin α1 on the immune system allows different immunomodulating effects depending on the immunological status of the recipient, a feature that could be extremely valuable in the context of COVID-19 [38]. Indeed, as already described in the first paragraph, the clinical course of the disease seems characterized both by immune disfunction/exhaustion and by paradoxical immune-hyperactivation (cytokine storm), with possibly unpredictable and very rapid switch from an initial phase of immunosuppressed status and a subsequent uncontrolled inflammatory reaction, characterizing the pathogenesis of the pulmonary damage [1]. The pleiotropic actions of thymosin α1 could effectively buffer the pathogenetic mechanisms of different phases, especially if combined with trivial immunosuppressant, such as corticosteroids, in the late stage of COVID-19.

Another element supports the possible role of thymosin α1 in controlling SARS-CoV-2 infection and severity. Indeed, its maintained production in the functional thymus of children may be responsible for their decreased susceptibility to COVID-19 [39]. The other way around, the severity of the disease among the elderly patients may be favored by the dramatic thymic involution, which has already been suggested as possibly responsible for the age-associated failure of the adaptive immune system [40].

This immunological background suggests the possible use of thymosin α1 in clinical trials for preventing COVID-19 severe evolution, as prophylaxis for SARS-CoV-2 infection, or as adjuvant for the vaccines currently undergoing experimental development.

Recently, a retrospective case series of 76 patients with severe COVID-19 was reported [41]: 36 were treated with thymosin α1, showing a significantly reduced mortality compared with 40 COVID-19 patients not receiving this treatment (mortality 11 vs 30%, respectively; p = 0.044). COVID-19 patients with low lymphocyte count gained more benefits from the drug, even in aged patients. Moreover, the authors showed that thymosin α1 significantly enhances T-cell counts in COVID-19 patients with severe lymphocytopenia and suggested its ability of reversing T-cell exhaustion, recovering immune reconstitution through promoting thymus output during SARS-CoV-2 infection. Indeed, they showed that thymosin α1 effectively downregulates both PD 1 and Tim 3 on CD8^+^ T cells in COVID-19 patients [41].

Subsequent evidence, even stronger, came from a multicenter retrospective analysis of 334 COVID-19 patients enrolled to receive thymosin α1 for 5 days or more from December 2019 to March 2020 [42]. The primary outcomes measured were the 28- and 60-day mortality; the secondary outcomes were hospital length of stay and the total duration of the disease. The 28-day mortality between the thymosin α1 and nonthymosin α1-treated groups was significantly different in adjusted model (p = 0.016). According to the subgroup analysis, thymosin α1 therapy significantly reduced 28-day mortality (hazard ratio, HR: 0.11; 95% CI: 0.02–0.63; p = 0.013) by improving Pa0_2_/FiO_2_ ratio (p = 0.036) and prolonged the hospital length of stay (p = 0.024) as well as the total duration of the disease (p = 0.001) in the critical type patients (who were defined by age, blood parameters, acute physiology and chronic health evaluation) [42].

**Figure 1.**
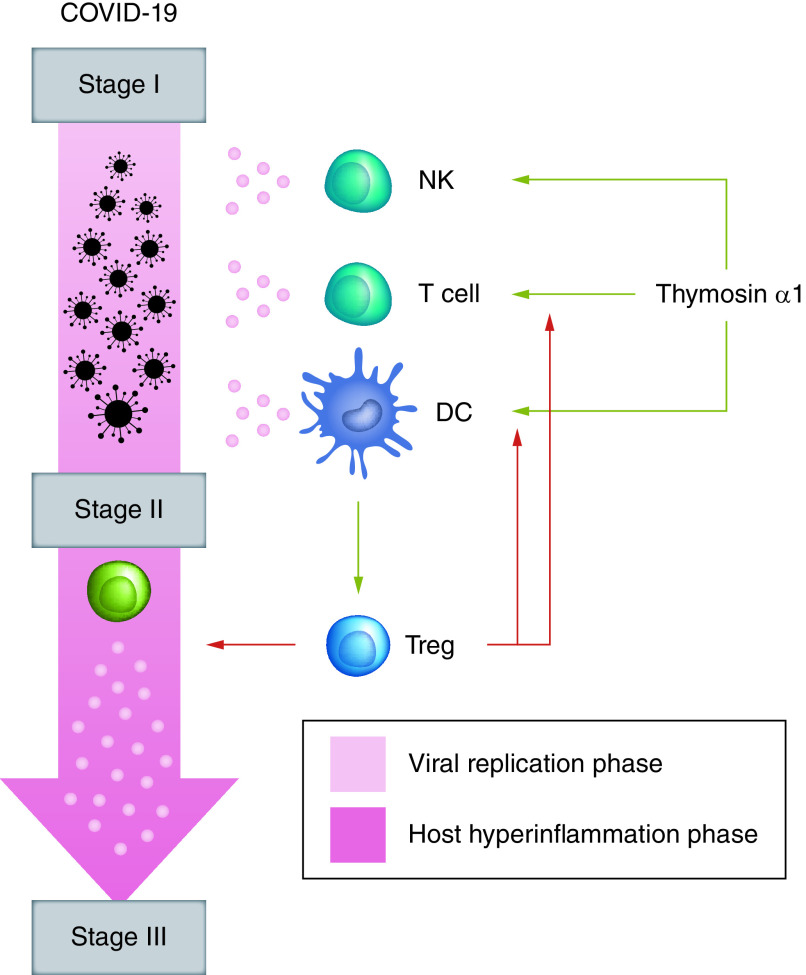
Proof of concept for thymosin α1 use in COVID-19. Thymosin α1 might have the ability to modulate the immune response to SARS-CoV-2 infection in the viremic phase, influencing the activity of T cells, NK lymphocytes and dendritic cells, modulating their cytokine production and avoiding progression into cytokine storm, thus preventing the evolution in severe forms of COVID-19. NK: Natural killer; SARS-CoV-2: Severe acute respiratory syndrome coronavirus 2.

As an immunomodulatory agent, thymosin α1 has several features rendering this drug a potential optimal candidate for a prophylactic approach to COVID-19 evolution. First, it is very well-tolerated, and any drug–drug interaction has never been reported, even in the case of anticancer drugs, to which have been associated in several trials as immune adjuvant. Second, its pleiotropic function allows different immunomodulating effects depending on the immunological status of the recipient, a feature that could be crucial in the context of COVID-19. The peculiar ability of thymosin α1 to restore immune system homeostasis, renders this drug potentially able to contrast the etiopathogenesis of the damage, thus preventing the COVID-19 progression from the viremic phase to the more severe stages, as we represented in Figure 1.

## Conclusion

With the premises described above, we hypothesized the effectiveness of a prophylactic intervention with thymosin α1 in reducing the critical evolution COVID-19 number in high risk populations, preventing hospitalization and death from SARS-CoV-2 infection, reducing the development and the severity of COVID-19 complications. Such experimental application would be warranted among particularly frail population of individuals (such as elderly and cancer patients), especially in the light of the current drop in new infections. Among the frailest patient categories, cancer patients are both at high risk of exposure and of lethal complications. Recent studies demonstrated that patients affected by cancer might have a higher risk of COVID-19, and showed poorer outcomes, than individuals without cancer [43–47]. A prophylactic approach would be warranted for these frail patients during the ‘Phase III’, in order to reduce the critical evolution of COVID-19, preventing hospitalization and death, minimizing the anticancer treatment discontinuation rate and consequently improving the oncological outcome.

## Future perspective

On the bases described above, we have designed the PROTHYMOS trial, a multicenter, open-label, Phase II randomized study (Eudract no. 2020-006020-13), with the primary objective of evaluating the efficacy of thymosin α1 as prophylaxis of serious COVID-19, defined as COVID-related hospitalization or death, in cancer patients undergoing active treatment (including neoadjuvant, adjuvant and advanced settings, with chemotherapy, immunotherapy or targeted therapy).

The prophylaxis impact against COVID-19, as the primary objective of the study, will be evaluated in terms of incidence of serious COVID-19 within 8 weeks from randomization, comparing the experimental Arm versus the control Arm, as the primary end point.

Cancer patients represent a large subgroup at high risk of developing coronavirus infection and its severe complications. While other chronic disease can be followed at home, with phone assistance and with the aid of the general practitioner, anticancer treatments often require high intensity of care, with frequent hospital access and a great number of clinical controls, blood and radiological testing and physical examinations. As a consequence, cancer patients are at high risk of SARS-CoV-2 contagion. In addition, given their likely impaired immunological status, due to the oncological disease and exposure to immunosuppressant treatments (such as chemotherapy or radiotherapy), they are at high risk of severe consequences from COVID-19.

Thus, the primary objective of this study is preventing hospitalization and death from SARS-COV-2 infection, and the secondary aim is minimizing the anticancer treatment discontinuation rate in cancer patients due to COVID-19, consequently improving their outcome.

In order to prioritize the health interventions postponed during the emergency, constructing actions for the improvement of the postemergency management of frail patients, the specific secondary objectives are represented by the compliance to anticancer therapy, by the dose-intensity maintenance, by the evaluation of the hematologic toxicity of the anticancer treatment (especially leukopenia and lymphopenia). The secondary end points that will be specifically investigated are represented by the anticancer therapy discontinuation rate, the dose reduction/delay rate, the incidence of hematologic toxicity (especially in terms of leukopenia and lymphopenia) and the rate of adverse events.

Among cancer patients with advanced disease, representing the majority of the oncological patient population, the secondary aim of the study will be represented by the evaluation of possible benefit from thymosin α1 adjuvant use also in terms of improving the patient outcome. The outcome of interest, considered for the patient subgroup with metastatic/advanced disease (constituting the majority of cancer patients at our institutions), will be investigated in terms of anticancer treatment safety, objective response rate, progression-free survival, time to anticancer-treatment failure and cancer-specific overall survival. The hypothesis of an effective prophylactic approach to COVID-19 would have immediate clinical relevance, especially given the lack of curative approaches for the disease. Moreover, in the ‘COVID-19 vaccine race era’ both clinical and biological results coming from the PROTHYMOS trials could even support the rationale for future combinatorial approaches trying to raise vaccine efficacy in frail individuals. The expected impact on the public health is due to the reduction of deaths and hospitalizations from COVID-19, allowing savings healthcare resources, especially targeting the intervention in a frail population, as cancer patients, often requiring hospitalization in case of COVID-19 infection, due to their high risk of complications. Moreover, an effective secondary prevention of COVID-19 morbidity and lethality among cancer patients would reduce the heavy impact of the pandemic on the curability of oncological diseases, recently penalized in terms of health resources and at risk to be burdened by diagnostic and treatment delays in the endemic areas.

**Executive summary**

- COVID-19 is characterized by different stages from the viremic phase to the cytokine storm.
- The first phase is probably allowed by immune suppression, while the late stage is often characterized by paradoxical excess of immune activation, with a cytokine storm.
- Thymosin α1 is an immune-modulating agent drug with pleiotropic properties.
- Thymosin α1 might have the ability to positively modulate the immune response in the viremic phase of COVID-19, avoiding progression into cytokine storm.
- Prior experience suggested that the combination of thymosin α1 with anticancer therapies may improve the safety and the efficacy outcomes.
- In the context of SARS-CoV-2 pandemic, thymosin α1 could be investigated as prophylaxis of severe COVID-19 in cancer patients undergoing active therapy.
